# Phylogenetic supertree and functional trait database for all extant parrots

**DOI:** 10.1016/j.dib.2019.103882

**Published:** 2019-03-30

**Authors:** Kevin R. Burgio, Katie E. Davis, Lindsay M. Dreiss, Laura M. Cisneros, Brian T. Klingbeil, Steven J. Presley, Michael R. Willig

**Affiliations:** a Department of Ecology and Evolutionary Biology, University of Connecticut, 75 N. Eagleville Rd. U-3043, Storrs, CT, 06269, USA; b Institute of the Environment and Center for Environmental Sciences and Engineering, University of Connecticut, 3107 Horsebarn Hill Road, Storrs, CT, 06269, USA; c Department of Biology, University of York, Wentworth Way, York, YO10 5DD, UK; d Department of Geography, Middlebury College, 287 Bicentennial Way, Middlebury, VT 05753, USA; e Department of Natural Resources and the Environment, University of Connecticut, 1376 Storrs Road, Storrs, Connecticut 06269, USA

## Abstract

We present a complete dataset from the literature on functional traits including morphological measurements, dietary information, foraging strategy, and foraging location for all 398 extant species of parrots. The morphological measurements include: mass, total length, wing chord, culmen length, tarsus length, and tail length. The diet data describe whether each species is known to consume particular food items (e.g. nectar, berries, and carrion), foraging strategy data describes how each species captures or accesses food, and foraging location data describe the habitat from which each species finds food (e.g. ground, canopy, and subcanopy). We also present a time-calibrated phylogenetic supertree that contains all 398 extant species as well as 15 extinct species (413 total species). These data are hosted on the Figshare data depository (https://figshare.com/s/6cdf8cf00793deab7ba6).

**Table:** Specifications table

Subject area	*Ecology, biogeography, evolutionary biology*
More specific subject area	*macroecology, community ecology, functional ecology, biodiversity science, phylogenetics*
Type of data	*Functional trait database, phylogenetic supertree*
How data was acquired	*Literature review*
Data format	*Raw*
Experimental factors	*Trait data about the size, diet, and foraging strategy for all 398 extant parrots were compiled from the literature and the phylogenetic supertree was synthesized from existing molecular studies in the literature.*
Experimental features	*Functional trait data and a phylogenetic supertree for all extant parrot species is important in determining global conservation priorities for one of the most at-risk orders of birds.*
Data source location	*Figshare:*https://doi.org/10.6084/m9.figshare.5877324
Data accessibility	*Functional trait data and phylogenetic supertree and supporting data deposited*https://figshare.com/s/6cdf8cf00793deab7ba6
Related research article	*E. Kosman, K.R. Burgio, S.J. Presley, M.R. Willig, S. Scheiner, In press. Conservation prioritization based on trait-based metrics illustrated with global parrot distributions. Divers. Distrib.*

**Table:** 

**Value of these data** • These data will be useful to ecology (e.g. evaluating community assembly at multiple spatial scales), conservation biology (e.g. identifying hotspots of functional or phylogenetic biodiversity), and macroecology, especially that of parrots. • The supertree will be a valuable resource to advance understanding of parrot evolutionary history and diversification. • These data can be used to explore various aspects of functional and phylogenetic diversity, as well as topics concerning functional and phylogenetic distinctiveness within communities as a tool to aid in conservation prioritization.

## 1 Data

**Fig. 1:**
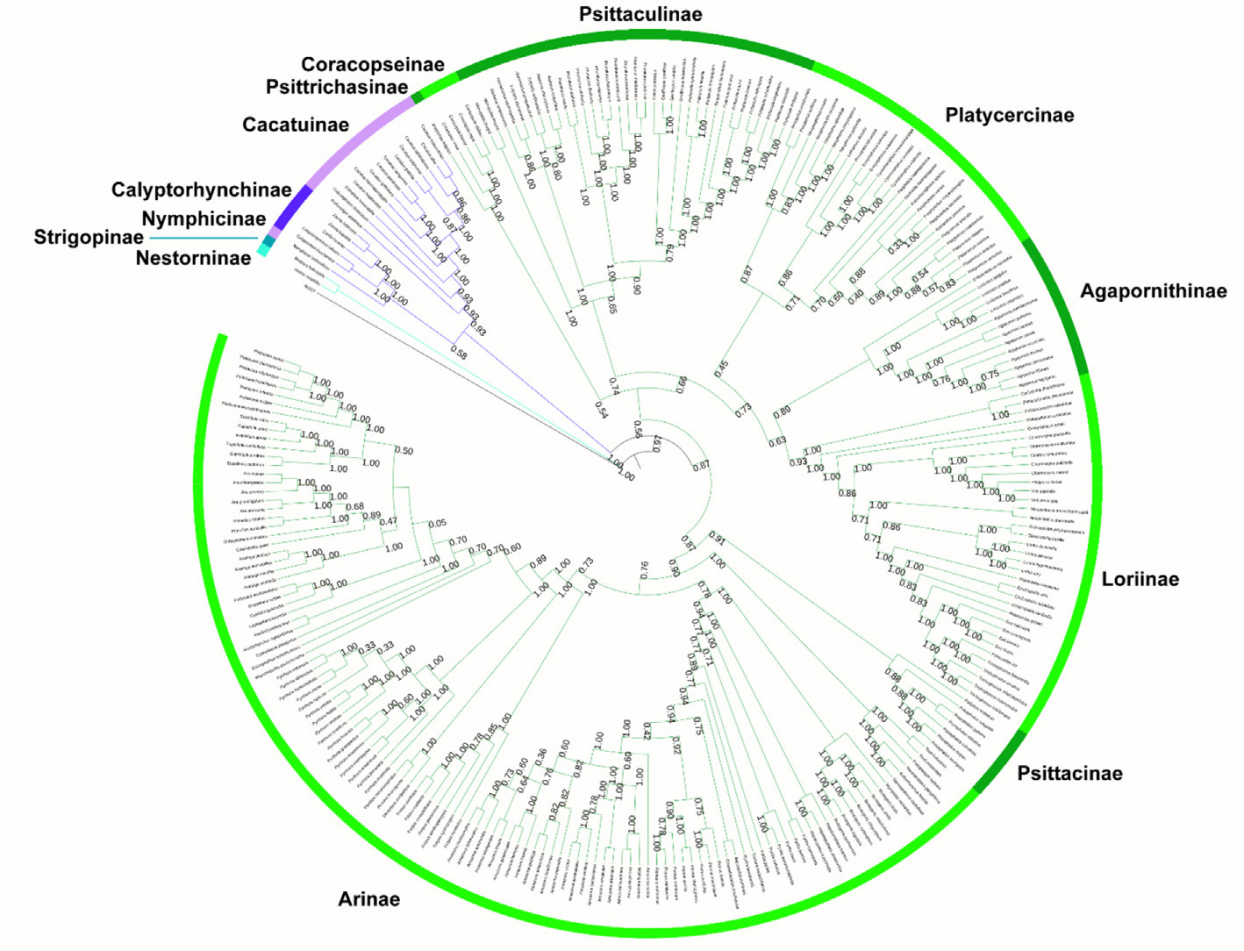
Maximum agreement subtree (MAST) supertree with V+ node support values. Green, purple, and blue indicate members of the Psittacidae, Cacatuidae, and Strigopidae, respectively, compromising all of the order Psittaciformes.

**Fig. 2:**
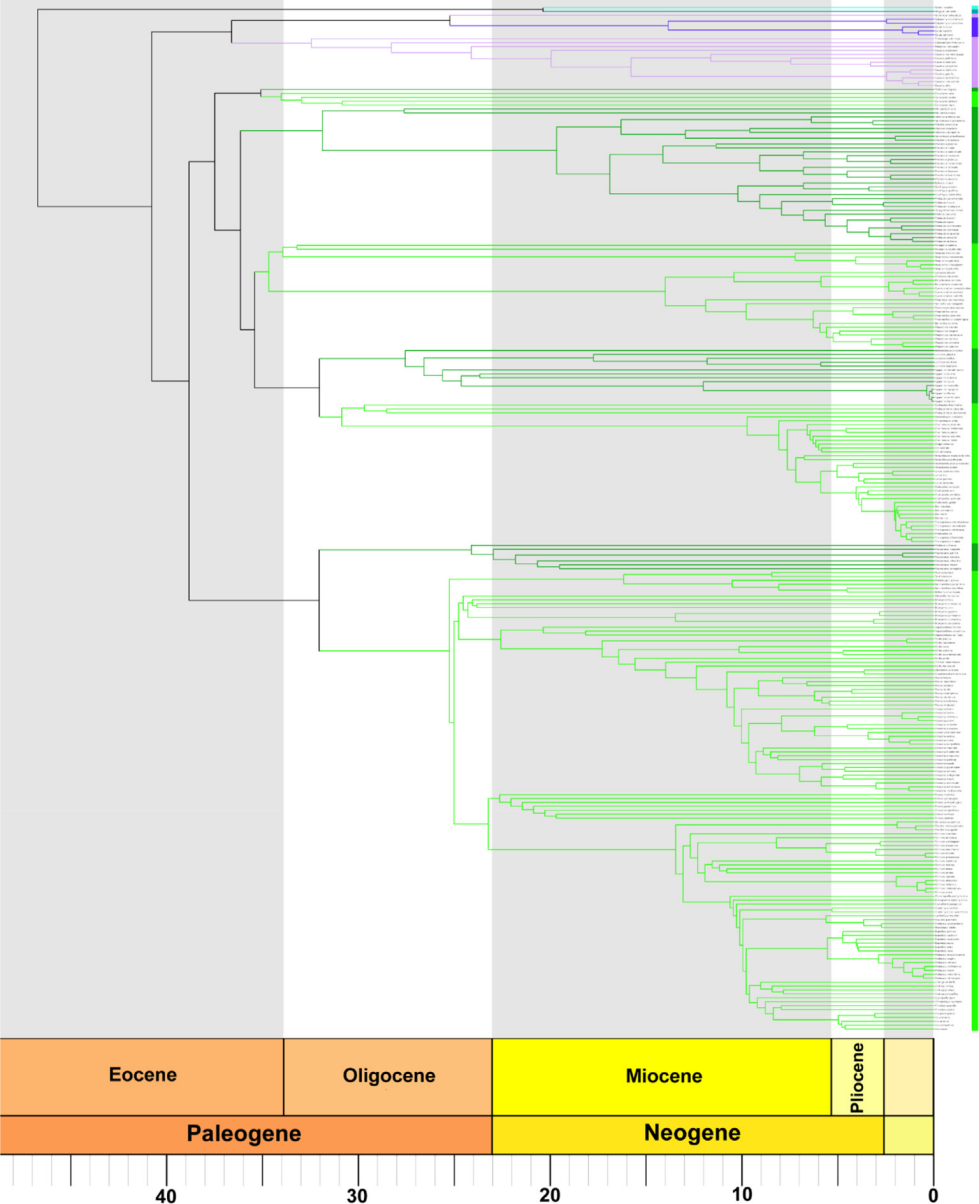
Complete time-calibrated supertree plotted with respect to the geological time scale using the R package ‘strap’ [29]. As in Fig. 1, green, purple, and blue indicate members of the Psittacidae, Cacatuidae, and Strigopidae, respectively, compromising all of the order Psittaciformes.

We present a trait database and a phylogenetic supertree for all 398 extant and 15 extinct parrot species. The trait database is a spreadsheet that includes measurements of various morphological characteristics and data on diet, foraging strategy, and foraging location, which are key traits to assess each species’ role within a community (Table 1). The supertree includes: two text files of all source data included in the analysis, a text file of the Matrix Representation with Parsimony (MRP) matrix used to construct the supertree; a spreadsheet of the node calibration data used for time-calibration of the tree, and the final time-calibrated complete species-level supertree in Newick format (see Fig. 1, Fig. 2). The data presented in this paper were used for analyses in Kosman et al. [1].

**Table 1:** Functional attributes included in the dataset. Data type: Categorical. Each trait is coded as either a “ 1” or a “0” depending on if the species is known to exhibit the attribute. For di et species known to eat a specific food item are coded with a “ 1” and species not known to each that specific food item are coded with a “ 0”. Similarly, for foraging strategy and location, species known to forage a certain way and in certain parts of the ecosystem were coded with a “ 1” and species not known to use such foraging strategies or locations were coded with a “ 0”. Data type: Mensural. Values are the average measurement for each of attribute of body size as indicated, measured from museum specimens or gleaned from the literature [2], [3], [4], [5].

Type of data	Functional component	Attribute	Trait values
Categorical	Diet	Carrion	1, 0
		Invertebrates	1, 0
		Snails	1, 0
		Pollen	1, 0
		Nectar	1, 0
		Flower	1, 0
		Seed	1, 0
		Nut	1, 0
		Fruit	1, 0
		Plant matter	1, 0
		Roots	1, 0
		Fungi	1, 0
	Foraging Strategy	Glean	1, 0
		Dig	1, 0
		Scavenge	1, 0
		Graze	1, 0
		Flower probe	1, 0
		Excavate	1, 0
	Foraging Location	Water	1, 0
		Ground	1, 0
		Vegetation	1, 0
		Subcanopy	1, 0
		Canopy	1, 0
Mensural	Body Size	Mass	Mean (g)
		Length	Mean (cm)
		Tarsus	Mean (mm)
		Culmen	Mean (mm)
		Wing	Mean (mm)
		Tail	Mean (mm)

## 2 Experimental design, materials and methods

### 2.1 Functional trait data

We obtained trait data from the literature for all parrot species [2], [3], [4], [5], following the taxonomy of del Hoyo et al. [6]. Categorical attributes include components of diet, foraging strategy, and foraging location, whereas mensural attributes characterize various body dimensions (see Table 1) The diet data describe whether each species is known to consume particular food items (e.g. nectar, berries, and carrion), foraging strategy data describes how each species accesses food, and foraging location describes the where in the habitat each species finds food (e.g. ground, canopy, and subcanopy). We used a similar method of characterizing the functional traits of each species, as well as addressing missing data for each species as [7], [8]. In total, we estimated only ∼4.8% of trait values (452 of 11,090) by linear regression or by substituting values from closely related taxa. Spreadsheets of the raw dataset both with and without inferred values can be found on Figshare: https://figshare.com/s/6cdf8cf00793deab7ba6.

### 2.2 Phylogenetic supertree

Supertrees exist for many taxa, such as dinosaurs [9], [10], birds [11], mammals [12], crocodiles [13], crustacean decapods [14], [15], [16] and grasses [17]. They allow fast and efficient synthesis of existing phylogenetic information (known as source trees), without the resources required for constructing complete molecular phylogenies [18]. Because there is no complete, up to date, phylogeny currently available for all parrot species, we created a new MRP (Matrix Representation with Parsimony) supertree for the order Psittaciformes.

We collected data via a thorough literature search and followed the processes and methodology as described in detail in Davis & Page [11] and Davis et al. [14] and all data processing was carried out using the Supertree Toolkit software [19]. The final data set was analysed with parsimony in TNT [20]. We used the xmult command incorporating a round of TBR branch breaking along with multiple parameters aimed at maximising the chances of finding the shortest trees, including running multiple replications, using sectorial searches, drifting, ratchet and fusing combined. Each replicate held 1000 trees for ratchet/drifting/rebuilding (the default is 1) and we ran 1000 independent replicates, each with a different (random) starting point. As these replicates were run independently, not in true parallel, we checked that the shortest trees were unique. The consensus tree(s) were then computed from the shortest unique MPTs. This resulted in 960 Most Parsimonious Trees (MPTs) of length 3150 steps. We then computed a Maximum Agreement Subtree (MAST) using PAUP* [21] to remove conflicting leaves. This resulted in a fully bifurcating supertree containing 273 of 413 parrot species (69%). There were no misplaced taxa or misleading relationships, as may sometimes happen with the MRP method [22], [23], recovered by the analysis. No novel clades were found in this analysis. Node support was calculated using the V+ index [24]. All support values for the supertree were positive nodes, and the vast majority had support of at least 75% (Fig. 1).

A complete species tree was necessary to enable analyses of phylogenetic biodiversity. Since the resulting supertree contained only 69% of extant species, we used an algorithm based on classification to add the remaining taxa to the tree. As a conservative measure, taxa were placed according to their least inclusive known taxonomy. Taxonomy levels went from family to subfamily to tribe (where applicable), and to genus. A taxon not already in the supertree was placed at the base node of the least inclusive clade that the taxon was known to be part of according to classification. For example, where a taxon A is known to be a member of subfamily A, according to taxonomy, taxon A would be placed at the node that was the MRCA of all taxa in the supertree included in subfamily A. If multiple taxa were to be placed at the same node a polytomy was created. We followed the del Hoyo et al. [6] classification for all taxonomic placements of taxa. We added additional species as polytomies, which reduced the resolution of the supertree but facilitated further analyses using a complete species-level tree of Psittaciformes.

Next, we time-calibrated the supertree. External data are required to time scale parsimony trees. Generally, fossil age data and dates of geological events are used to assign dates to nodes in the tree after which various algorithms can be employed to extrapolate dates for the remaining nodes. Parrots have a poor fossil record and published molecular phylogenies have used external fossil calibration points from outside Psittaciformes [25], [26], [27]. Molecular analyses run with a molecular clock produce fully time-calibrated trees; therefore, we used node dates from these published phylogenies by applying them to any nodes shared by both the molecular tree(s) and our supertree. We allocated dates for the remaining nodes in the supertree using the R package “paleotree” [28]. We chose the “equal” method with the minimum branch length set 0.1 Myr, resulting in a fully time-calibrated supertree used to estimate phylogenetic diversity (Fig. 2). See “ParrotNodeDates” on Figshare for node numbers and calibration dates, and “ParrotSuperTree” on Figshare for the complete time-calibrated supertree in Nexus format.
